# Independent component analysis: a reliable alternative to general linear model for task-based fMRI

**DOI:** 10.3389/fpsyt.2023.1214067

**Published:** 2023-08-16

**Authors:** Kostakis Gkiatis, Kyriakos Garganis, Irene Karanasiou, Athanasios Chatzisotiriou, Basilios Zountsas, Nikolaos Kondylidis, George K. Matsopoulos

**Affiliations:** ^1^School of Electrical and Computer Engineering, National Technical University of Athens, Athens, Greece; ^2^Epilepsy Monitoring Department, St. Luke's Hospital, Thessaloniki, Greece; ^3^Department of Mathematic and Engineering Sciences, Hellenic Military Academy, Athens, Greece; ^4^Department of Neurosurgery, St. Luke's Hospital, Thessaloniki, Greece; ^5^Department of Physiology, Medical School Aristotle University of Thessaloniki, Thessaloniki, Greece; ^6^Radiological Department, St. Luke's Hospital, Thessaloniki, Greece

**Keywords:** language mapping, fMRI, ICA, GLM, presurgical evaluation, neuroimaging, brain mapping, epilepsy

## Abstract

**Background:**

Functional magnetic resonance imaging (fMRI) is a valuable tool for the presurgical evaluation of patients undergoing neurosurgeries. Although many pre-processing steps have been modified according to advances in recent years, statistical analysis has remained largely the same since the first days of fMRI. In this study, we examined the ability of Independent Component Analysis (ICA) to separate the activation of a language task in fMRI, and we compared it with the results of the General Lineal Model (GLM).

**Methods:**

Sixty patients undergoing evaluation for brain surgery due to various brain lesions and/or epilepsy and 20 control subjects completed an fMRI language mapping protocol that included three tasks, resulting in 259 fMRI scans. Depending on brain lesion characteristics, patients were allocated to (1) static/chronic not-expanding lesions (Group 1) and (2) progressive/expanding lesions (Group 2). GLM and ICA statistical maps were evaluated by fMRI experts to assess the performance of each technique.

**Results:**

In the control group, ICA and GLM maps were similar without any superiority of either technique. In Group 1 and Group 2, ICA performed statistically better than GLM, with a *p*-value of < 0.01801 and < 0.0237, respectively. This indicated that ICA performs as well as GLM when the subjects are able to cooperate well (less movement, good task performance), but ICA could outperform GLM in the patient groups. When both techniques were combined, 240 out of 259 scans produced reliable results, showing that the sensitivity of task-based fMRI can be increased when both techniques are integrated with the clinical setup.

**Conclusion:**

ICA may be slightly more advantageous, compared to GLM, in patients with brain lesions, across the range of pathologies included in our population and independent of symptoms chronicity. Our findings suggest that GLM analysis may be more susceptible to brain activity perturbations induced by a variety of lesions or scanner-induced artifacts due to motion or other factors. In our research, we demonstrated that ICA is able to provide fMRI results that can be used in surgery, taking into account patient and task-wise aspects that differ from those when fMRI is used in research.

## 1. Introduction

Functional magnetic resonance imaging (fMRI) has proven to be a valuable tool for the non-invasive presurgical evaluation of patients undergoing brain surgery in regions near the eloquent cortex. Task-based fMRI utilizes carefully designed tasks related to neuropsychological processes and the statistical analyses that follow in the processing pipeline to activate the brain regions relevant to motor, language, memory, and other functions. Most fMRI studies focus on group analysis of tasks to reveal common functional networks in the population. Nevertheless, the true limitations of fMRI analysis techniques are in subject-specific analysis, which is necessary for a clinical setup.

As far as pre-processing of fMRI data is concerned, many improvements have been made in recent decades. Moreover, motion artifact correction (1–3), slice time correction (4, 5), spatial smoothing (6–9), and registration (10–12) have been extensively studied, all of which demonstrate a high signal-to-noise ratio of pre-processed fMRI datasets.

In clinical setups and scientific experiments, the General Linear Model (GLM) is the most used statistical analysis. As a statistical tool, GLM has its intrinsic limitations in addition to the fMRI experiment-specific complexities (13, 14). Movement during the scan, instabilities in the scanner field, or the inability of the subject to perform the task correctly throughout the fMRI experiment can render the design matrix inappropriate and, thus, the results inaccurate. Furthermore, it is debatable whether the same design matrix is suitable for different brain regions as the hemodynamic response differs across the brain. Especially, if the subject's movement is in accordance with the experimental design, then the movement regressors will be correlated with the task regressor, leading to erroneous beta estimations. Another problematic aspect is the linearity that GLM assumes, given that the dynamics within the brain are known to be far from linear.

In psychiatric fMRI experiments, the strength of the activations is important and may vary in accordance with many neuropsychological aspects, such as the level of attention, medication, or even the level of task familiarization of the subject. Such experiments are usually more sophisticated in their designs with complex scientific hypotheses where the individual variability may be significant. These factors cannot be modeled in the design matrix, and single-subject GLM analysis is, therefore, not appropriate for drawing patient-specific conclusions (15). As such, these fMRI experiments have remained only in scientific protocols without the ability to move to clinical setups, as GLM analysis can only produce reliable results in group analysis.

Although the scientific community is aware of these shortcomings, the statistical analysis process using GLM of single subjects has mostly remained unchanged since the first days of fMRI. Independent component analysis (ICA) has been proposed to overcome these shortcomings. ICA is a data-driven exploratory technique that searches independent spatial distributed maps that can explain the captured signal. The technique was first introduced to fMRI data analysis in 1998 (16) and has since been established in the field (17). ICA has been successfully applied in many studies, mainly in the form of group ICA analysis as well as in the form of simple and controlled tasks to prove the validity of ICA.

In the present study, we examine the ability of ICA to extract the fMRI activations in an uncontrolled clinical environment and in a sophisticated language task protocol that activates multiple and distant brain regions, and we compare the results with those of the respective GLM analysis. We included all patients that underwent language task fMRI studies in our hospital for the duration of the study. We avoided setting any exclusion criteria to assess the effectiveness of ICA in a real clinical setup. For our results to be clinically meaningful and applicable to the most common populations brought to brain surgery, we allocated patients to two major groups: Group 1, consisting of patients with chronic/static and not-expanding brain pathology, manifesting mainly with epileptic seizures, and Group 2, consisting of patients with progressive brain pathology, mostly malignant brain tumors manifesting with epileptic seizures and, perhaps, other neurologic symptoms as well. We also included a control group (CG) to assess the differences between the two techniques in a supervised environment. We hypothesized that the two techniques would be equally sufficient and would supplement each other in a clinical setup.

## 2. Material and methods

### 2.1. Study participants

The current study included healthy volunteers as well as patients undergoing presurgical evaluation prior to brain surgery. All data were acquired from July 2018 to January 2022 in St. Luke's Hospital, Thessaloniki, Greece. Twenty healthy volunteers were recruited for the evaluation of the fMRI task protocol (18) and included in this study as the control group. All healthy subjects provided written consent for their participation. The healthy volunteers performed all three tasks, resulting in 60 scans. Patients' data were selected retrospectively, all their imaging data were acquired in the context of their presurgical evaluation routine, and the protocols were not modified in any way for the current study. As this is a retrospective study performed in accordance with the Declaration of Helsinki, the ethical approval of the current study was waived by the institutional review board (IRB).

As the current study aimed to evaluate the ability of ICA to extract the activation component in task-based fMRI, no exclusion criteria were set, and all available data were used. The imaging data of 60 patients were available (mean age 31.3 ± 15.6 years; 29 women; Table 1). Due to a lack of cooperation from some patients, some tasks were performed twice while others could not be completed. Consequently, 199 scans from 60 patients were collected and analyzed. The 60 patients were further divided into two groups. Group 1 consisted of 38 patients with 130 scans, all with chronic epilepsy and the following brain lesions subgroups, as suggested by structural MRI findings and histopathology exams following surgery. These lesions were either congenital or early-life acquired.

- *N* = 18 patients with static/non-progressive developmental neoplasms (ganglioneuronal tumors and Grade I Glial Neoplasms). These are lesions associated with chronic intractable epilepsies. They are also known as long-term epilepsy associated tumors (LEAT).
- *N* = 3 patients with neurodevelopmental malformations (NDM). These are lesions usually due to genetically-determined aberrant structural brain organization and deficits.
- *N* = 10 patients with gliotic-scar lesions.
- *N* = 3 patients with medial temporal sclerosis (MTS), a particular atrophic-gliotic lesion of the medial temporal lobe structures.
- *N* = 4 patients with unknown pathology. Structural MRI clues indicating a possibly abnormal area often exist. However, documentation of a specific lesion is lacking unless the patient is brought to the surgery and the excised tissue is subjected to histopathologic analysis.

**Table 1:** Characteristics of participants (*N* = 80).

**Controls (*****N*** = **20)**		
Women, *n* (%)	11 (55)	
Age, mean in years (std, range)	31.6 (7.4, 18–44)	
Years of education, mean in years (std, range)	15.85 (2.18, 12–20)	
**Handedness**, ***N*** **(%)**		
Right	20 (100)	
Left	0 (0)	
**Language Lateralization**, ***N*** **(%)**		
Left	19 (95)	
Right	1 (5)	
**Patients (*****N*** = **60)**	**Group 1 (*****N*** = **38)**	**Group 2 (*****N*** = **22)**
Women, *n* (%)	18 (47.3)	11 (50)
Age, mean in years (std, range)	26.68 (13.7, 9–63)^*^	39.8 (16.07, 11–71)^*, +^
Years of education, mean in years (std, range)	11.58 (4.87, 2–21)^**, ++^	14.86 (3.62, 6–21)^**^
**Handedness**, ***N*** **(%)**		
Right	27 (71.1)^+^	18 (81.8)
Left	7 (18.4)	2 (9.1)
Bilateral	4 (10.5)	2 (9.1)
**Language lateralization**, ***N*** **(%)**		
Left	29 (76.3)	20 (91)
Right	6 (15.8)	1 (4.5)
Bilateral	3 (7.9)	1 (4.5)
**Affected hemisphere**, ***N*** **(%)**		
Left	28 (73.7)	20 (90.9)
Right	9 (23.7)	2 (9.1)
Unknown	1 (2.6)	0 (0)
**Pathologies**, ***N*** **(%)**		
LEAT	18 (47.4)	
NDM	3 (7.9)	
Gliosis	10 (26.3)	
MTS	3 (7.9)	
Unknown	4 (10.5)	
Grade II astrocytoma		4 (18.2)
Grade III astrocytoma		5 (22.7)
Grade IV astrocytoma		10 (45.5)
Rasmussen encephalitis		2 (9.1)
Metastatic brain tumor		1 (4.5)

^*^Statistical significance of a p-value of < 0.05 between Group 1 and Group 2, ^**^Statistical significance of a p-value of < 0.01 between Group 1 and Group 2, ^+^Statistical significance of a p-value of < 0.05 with the control group, ^++^Statistical significance of a p-value of < 0.01 with the control group.

Group 2 consisted of 22 subjects with 69 scans that presented with recent onset epileptic seizures as well as other neurologic symptoms, depending on lesion location. These lesions may appear across a wide age range of patients, progressively enlarge, invade the brain, and, as a result of this, manifest quite early after initiation of the pathologic process. This group consisted mainly of malignant brain tumors [Grade II (*N* = 4), Grade III (*N* = 5), and Grade IV (*N* = 10)], Rasmussen Encephalitis (*N* = 2), and one case of metastatic brain tumors (*N* = 1).

Finally, 259 scans (60 from healthy controls and 199 from patients) were used in the current study. All data were fully anonymized prior to any processing. Demographics of the cohort are shown in Table 1. Details about the control cohort can be found in Gkiatis et al. (18).

### 2.2. Task fMRI protocol

Details about the language fMRI task protocol that was implemented can be found in Gkiatis et al. (18) and Benjamin et al. (19). In brief, the protocol includes three lexico-semantic tasks. In the first task (Object Naming–ON), drawn objects were presented to the subjects, and they were instructed to silently name each object and an action that could be performed with it (20). In the second task (Verbal Responsive Naming–VRN), written descriptions of concrete nouns were presented to subjects, and they were instructed to silently name the described object (21). In the last task (Auditory Responsive Naming – ARN) (21, 22), auditory-cued descriptions of concrete nouns were presented to the subjects through headphones, and they were instructed to silently name the described object. All tasks consisted of 6 repetitions of 24 s for the task periods and 24 s for the control periods. Control periods were carried out according to each task to control for the stimulus-specific activations. Pre-scan training was performed by all participants until they were familiar with the tasks prior to any acquisition.

### 2.3. MRI scanner protocols

All MRI images were acquired in St. Luke's Hospital, Thessaloniki, Greece. A Siemens Avanto FIT 1.5T (Siemens Healthineers, Erlangen, Germany) MRI scanner was used. A standard Siemens 20-channel head and neck coil with simultaneous multi-Slice (SMS) capabilities was employed. Acquisitions began with a standard Siemens field mapping sequence: TR 1,010 ms, TE 4.76 ms, and 9.52 ms, flip angle 60°, voxel-size 2 mm^3^, and FoV 228 × 228 × 170 mm. After that, a 15-min fMRI resting state was acquired. Then, the three language fMRI tasks were carried out. The resting state fMRI and task fMRI scanner protocols were single-shot echo-planar imaging protocols with the same acquisition parameters: multi-band factor 4, TR 1,700 ms, TE 50 ms, flip angle 84°, FoV 204 × 204 × 120 mm, and voxel-size 2 × 2 × 2 mm in a plane matrix of 102 × 102 voxels. Following that, a 3D T1-weighted image with a magnetization-prepared rapid gradient-echo (MPRAGE) sequence was acquired for registration purposes with the following parameters: GRAPPA factor 2, TR 2,200 ms, TE 2.97 ms, TI 900 ms, flip angle 8°, FoV 250 × 250 × 192 mm, and voxel-size 1 × 1 × 1 mm in a plane matrix of 256 × 256 voxels, with axial acquisition. A 3D T2-weighted image was also obtained with the following parameters: GRAPPA factor 2, TR 5,000 ms, TE 335 ms, TI 1,800 ms, FoV 260 × 252 × 176 mm, a matrix of 256 × 248 voxels with a voxel-size of 1 × 1 × 1 mm, with sagittal interleaved acquisition. The resting state fMRI protocol included 530 volumes, while the task fMRI protocol included 177 volumes for each task. Data are not currently publicly available.

### 2.4. fMRI pre-processing

To avoid the introduction of unnecessary biases in the analysis, the same pre-processing steps were maintained for both analyses. Pre-processing steps were minimal to prevent unnecessary interpolations that could influence the results. All data were fully anonymized prior to any processing.

Pre-processing pipeline was implemented in FMRIB's Software Library (FSL; v 6.0.1; https://www.fMRIb.ox.ac.uk/fsl) (23). First, T1-weighted 1 mm^3^ isotropic images were skull-stripped with the optiBET tool—which outperformed all other tools when extensively tested in 70 patients' brain (24)—for the registration procedures. In the data from the fMRI scans, the first three volumes were discarded for the scanner's T1 signal stabilization purposes. The last four volumes were also discarded as they were added to ensure that no data would be lost and the task was completed during their acquisition. The remaining 170 volumes were used for the analysis. The middle image of the fMRI sequence was defined as a template. All volumes were registered to the template with a rigid body transformation with six degrees of freedom to correct for patients' head movement during the scan (3). The MRI scanner's B0 field inhomogeneities were estimated via the field map sequences that were acquired at the beginning of each session and were corrected accordingly after the registration of the maps to the template (25). FMRI data were brain extracted by estimating the best thresholding value to remove the skull while avoiding the removal of any brain tissue as it was implemented in the FSL toolbox. A Gaussian kernel of double the voxel-size (4 mm) full width at half maximum (FWHM) was selected and applied for spatial smoothing of the data ensuring high SNR while eliminating high abrupt peaks in the scans. A high-pass filter was applied with a cut-off frequency matching the task frequency at 50 s. Registration to the T1-weighted images was implemented by registering the template to this image with the highly adopted boundary-based registration (BBR) algorithm. After performing the rigid body transformation with 12 degrees of freedom, this algorithm implements slight corrections according to the boundaries of the white and gray matter (26).

### 2.5. Data analyses

The current study aimed to provide evidence on whether ICA can be a viable alternative to GLM analysis for analyzing task-based fMRI in the presurgical evaluation of patients. As such, we intended to test this hypothesis in real-world data, which may include noisy data with some subjects exhibiting excessive motion or not performing well due to the potential inability to cooperate caused by their neuropsychological state. In this context, (a) no exclusion criteria were set, and all subjects who underwent fMRI language mapping for their presurgical evaluation were included in the study, (b) the analyses were the same for all datasets irrespective of the noise level, and (c) all analyses were performed in the single subject level to extract the language map of each patient individually.

#### 2.5.1. GLM analysis

General Linear Model (GLM) analysis is a univariate statistical method that is widely used and has verified its efficacy in task-based fMRI independent of the task. GLM takes the form:


Y=Xβ+e,

where *Y* refers to the time series of the voxel being tested, *X* is the design matrix or, equivalently, the matrix of regressors of the fMRI model, *e* refers to the error of the model, and β are the parameters of the model that can be estimated as:


$$\hat{\beta }={\left(X^{T}X\right)}^{-\;1}X^{T}Y,$$

where the T sign denotes the transposed matrix, and −1 sign denotes the inverse matrix, and it should follow a Gaussian distribution with zero mean: *e*~ℕ(*O*, σ^2^*I*).

As such, the best estimation of *Y* is the ordinary least square (OLS):


$$\hat{Y}=X\overset{\wedge }{\beta }=X{\left(X^{T}X\right)}^{-1}X^{T}Y=P_{X}Y,$$

where $P_{X}=\;X{\left(X^{T}X\right)}^{-}X^{T}$, and the error *e* can be estimated as:


$$e=Y-Ŷ=Y-P_{X}Y=R_{X}Y,$$

where *R*_*X*_ = *I*−*P*_*X*_.

Though, the errors in an fMRI dataset are not independent of one another, and it follows a distribution: *e*~ℕ(*O*, σ^2^*V*), where *V* is the autocorrelation matrix of the time series that may have nonzero off-diagonal elements and different values across the diagonal illustrating cross-correlation and time-dependent differences in variance. As such, a pre-whitening stage was applied that involved the estimation of a matrix *W*, such that *WVW*^*T*^ = *I* or, equivalently, *V*^−1^ = *W*^*T*^*W*, with which the data are multiplied:


WY=WXβ+We,

which leads to an error: *We*~ℕ(*O*, σ^2^*I*) since the variance was: *Var*(*We*) = σ^2^*WVW*^*T*^ = σ^2^*I*

So, the model was now solved as follows:


$$\hat{\beta }={\left(X^{T}V^{-1}X\right)}^{-1}X^{T}V^{-1}Y$$

and


$$Ŷ=X\hat{\beta }=X{\left(X^{T}V^{-1}X\right)}^{-1}X^{T}V^{-1}Y$$

However, most importantly, the error has a normal distribution with zero mean. It is independent and identical, which can allow for correct model parameter $\hat{\beta }$ estimation. In our analyses, different σ^2^ of the error were assumed for each voxel to account for differences in brain activation as well as in noise level, which may reflect B0 inhomogeneities, BOLD signal discrepancies, or other factors in distant brain regions.

The creation of the model *X* in the GLM analysis is of high importance for the meaningful estimation of the parameter $\hat{\beta }$. The regressors of *X* can be divided into two categories: (a) regressors of interest and (b) nuisance regressors. In general, as regressors of interest, the time series of the task is chosen, while as nuisance regressors, the motion parameters and/or physiological measurements during the acquisition are chosen. In our analysis, since all fMRI scans were block-design, the regressor of interest was set:


$$X_{1}=\left(hrf^{*}f\right),$$

where ^*^ refers to the convolution of the two functions, *f* is a binary function with 1 s at the time that the task was performed and 0 s as the time when there was rest, and *hrf* is the model for the hemodynamic response function that was chosen for the current analyses, and it is composed of a single gamma function (std-dev: 3 s; mean lag: 6 s) after filtering it with a high-pass filter at 50 s. As nuisance regressors, the six motion parameters were chosen as well as the temporal derivative of *X*_1_ to account for shifts of *hrf* that may result due to slice time differences or hemodynamic response differences in different parts of the brain and/or between-subject variability of the hemodynamic response function. It should be noted that the derivatives of the motion parameters were not included in the model *X* in order to avoid excessive increases in the degrees of freedom.

#### 2.5.2. ICA analysis

Independent component analysis (ICA) is a multivariate blind source separation (BSS) method. The goal of ICA is to express the data, which are a set of random variables, as a combination of statistically independent non-Gaussian component variables, which are the signal sources, and it can be formulated by this simple matrix equation:


X=AS,

where *X* is the fMRI data rearranged into a *p*×*n*, with *p* being the time points of each voxel *n* in the dataset, *S* is the optimized *q*×*n* matrix containing the statistically independent spatial maps in each row, and *A* is the mixing *p*×*q* matrix containing the time course of each spatial map in its columns. The aim of the optimization algorithms is to estimate an unmixing matrix *W* = *A*^−1^ such that *S* = *WX* contains mutually independent rows. In the current study, probabilistic ICA (PICA) (17) was utilized. PICA also models additive Gaussian noise leading to:


X=AS+ η,

where η~ ℕ(*O*, σ^2^Σ). Similar to GLM analysis, a variance-normalization step takes place with a matrix *K*, such that *KΣK*^*T*^ = *I*, leading to a new error of $\bar{\eta }\sim \;\mathbb{N}\left(O,\;\sigma^{2}I\right)$ that follows the normal distribution with zero mean such that it is independent and identical.

Although the equations appear similar to that of GLM analysis, there are two major differences, (a) the matrix *A* is not predefined as in GLM, but rather it is estimated as part of the model fitting, and (b) ICA is a multivariate method, meaning matrix *X* and, subsequently, matrices *A* and *S*, that refers to the time series of the whole fMRI scan and not to the time series of a single voxel.

The next step in model fitting is the estimation of the dimensionality *q* of the matrix *A*, or, equivalently, the number of components to be extracted. To avoid manual annotation of the matrix dimensionality in each subject, we employed the Laplace approximation to the Bayesian evidence to estimate the model order (17) that estimates the value of *q* that maximizes the signal explained in the fMRI data while maintaining *q*<*p*. Finally, the unmixing matrix *W* was estimated in the space of the pre-whitened data and on the principle of non-Gaussianity and statistical independence of the source distribution. To convert each independent component that was calculated into a Z-score map that could be thresholded, the raw components were divided by the standard deviation of the estimated voxel-wise error η. The component of activation, or, equivalently, the ICA language map, was selected according to the structural distribution of the activations as well as the similarity to the GLM language map. In 6 out of the 259 fMRI scans, ICA split the activations into more than one component.

### 2.6. Experts' evaluation of language maps

Following the language maps, the GLM and ICA maps were thresholded and presented to two independent experts in language mapping with fMRI, GK, and KN, who reviewed and scored the maps accordingly.

#### 2.6.1. Language map thresholding

The language maps, independent of whether they were derived from GLM or ICA methodology, were thresholded with a combination of a fixed cluster-wise threshold at a *p*-value of < 0.05 and a voxel-wise threshold that ranged from 2.3 to 3.1 z-score (*p* < 0.01 to *p* < 0.0001). Four thresholded maps were generated for each methodology for each subject according to the voxel-wise threshold, one at 2.3 z-score, one at 2.6 z-score, one at 2.9 z-score, and one at 3.1 z-score.

#### 2.6.2. Evaluation procedure

**Figure 1:**
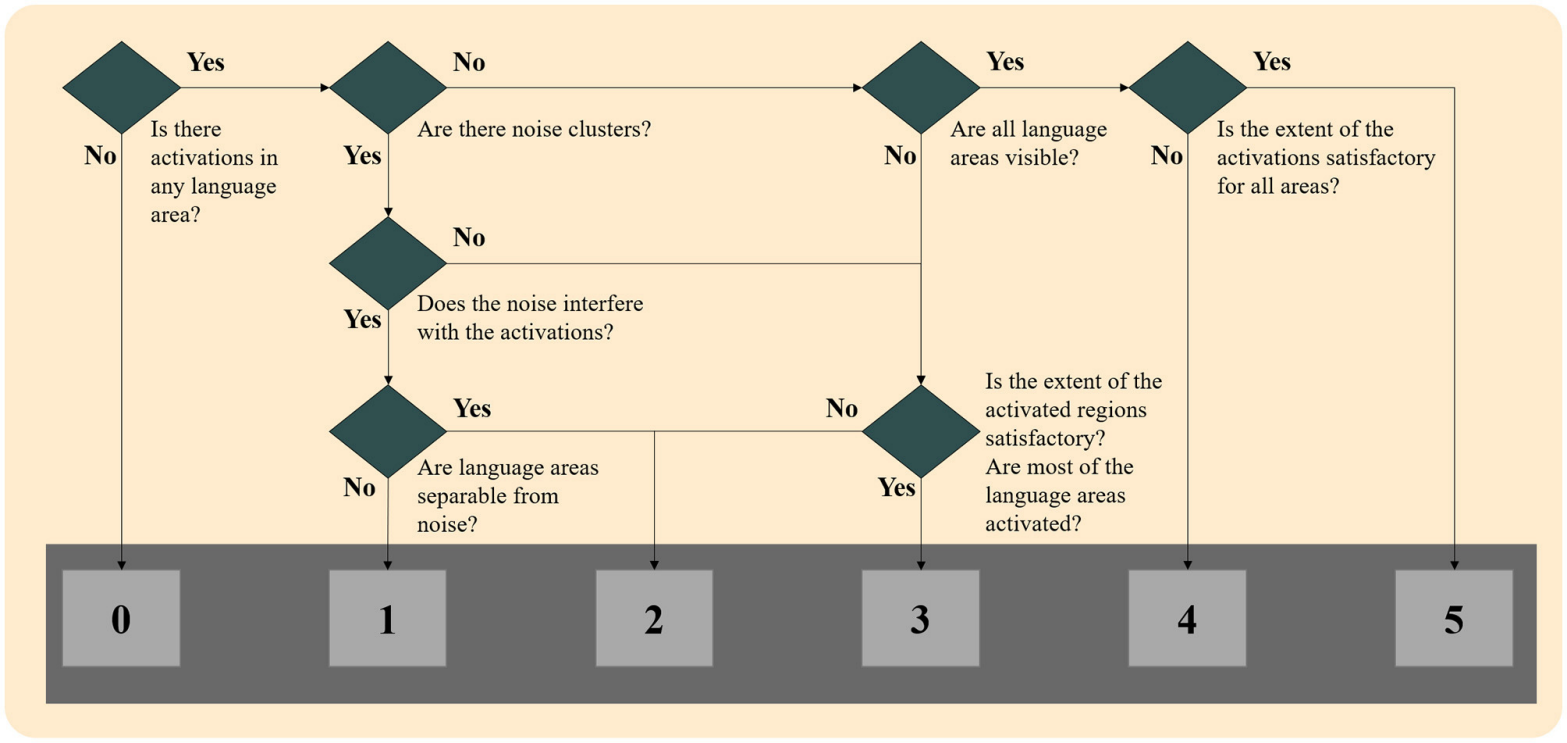
Flowchart of language map scoring for both techniques.

These four maps were presented to the experts blindly to whether the map was generated through GLM or ICA methodology, superimposed in the high-resolution space (T1-weighted images). The evaluation procedure is shown in Figure 1 and was as follows. First, they chose the most appropriate language map among the four thresholded maps of the specific methodology. Then, they scored the selected map ranging from 0 to 5 according to the following classification:

*0: No activations:* No cluster survived the thresholding, and/or noise clusters were visible, and none could be attributed to language-relevant regions.

*1: Unreliable activations:* Some clusters may be language activations, but noise clusters were interfering with these clusters, and no reliable conclusion could be made.

*2: Unreliable activations:* Only a few of the six-language critical regions were activated, and/or noise clusters were visible, and they interfered with the activation clusters.

*3: Somewhat reliable activations:* Activations in most of the six language-critical regions were visible, but some were missing, and/or noise clusters were visible without interfering with the activation clusters.

*4: Reliable activations:* Activations in all six language-critical regions were visible, but in some regions, the extent may not be satisfactory when compared to the mean of the control group. No noise clusters were visible.

*5: Reliable activations:* Activations in all six language-critical regions were visible and to a satisfactory extent when compared to the mean of the control group. No noise clusters were visible.

**Figure 2:**
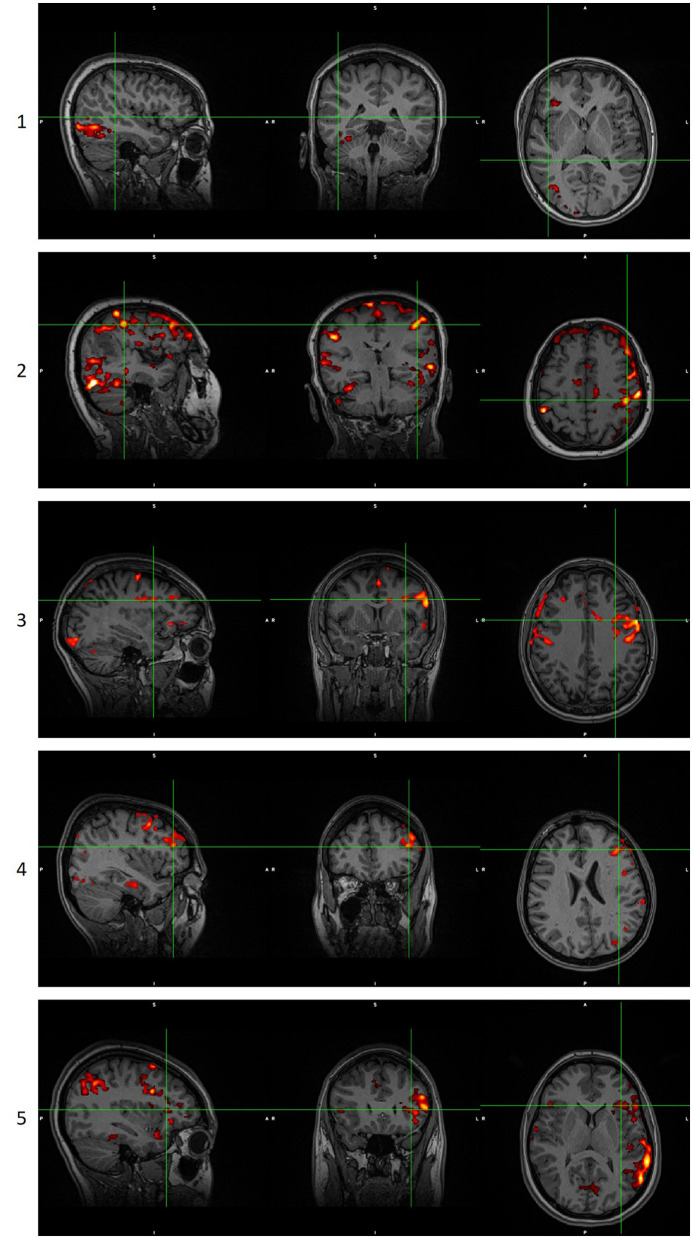
Examples of language maps for each score. Score 0 is not shown as it is a blank/noisy map. Images are in radiological orientation. GLM scoring when the score of ICA analysis was 0, 1, or 2. ICA scoring when the score of GLM analysis was 0, 1, or 2. GLM, General Linear Model. ICA, Independent Component Analysis.

When there was a disagreement between the scoring of the two experts, the two reviewers concurred on the final score according to the scoring table. When no cluster survived the thresholding in any methodology, maps were given a score of 0, and they were not presented to the reviewers. Examples of language maps for each score are presented in Figure 2. In the ICA methodology, in six maps where the activations were split into more than one component, the component that showed the most reliable activations was thresholded and shown to the reviewers, though they were still not informed of this procedure.

## 3. Results

**Figure 3:**
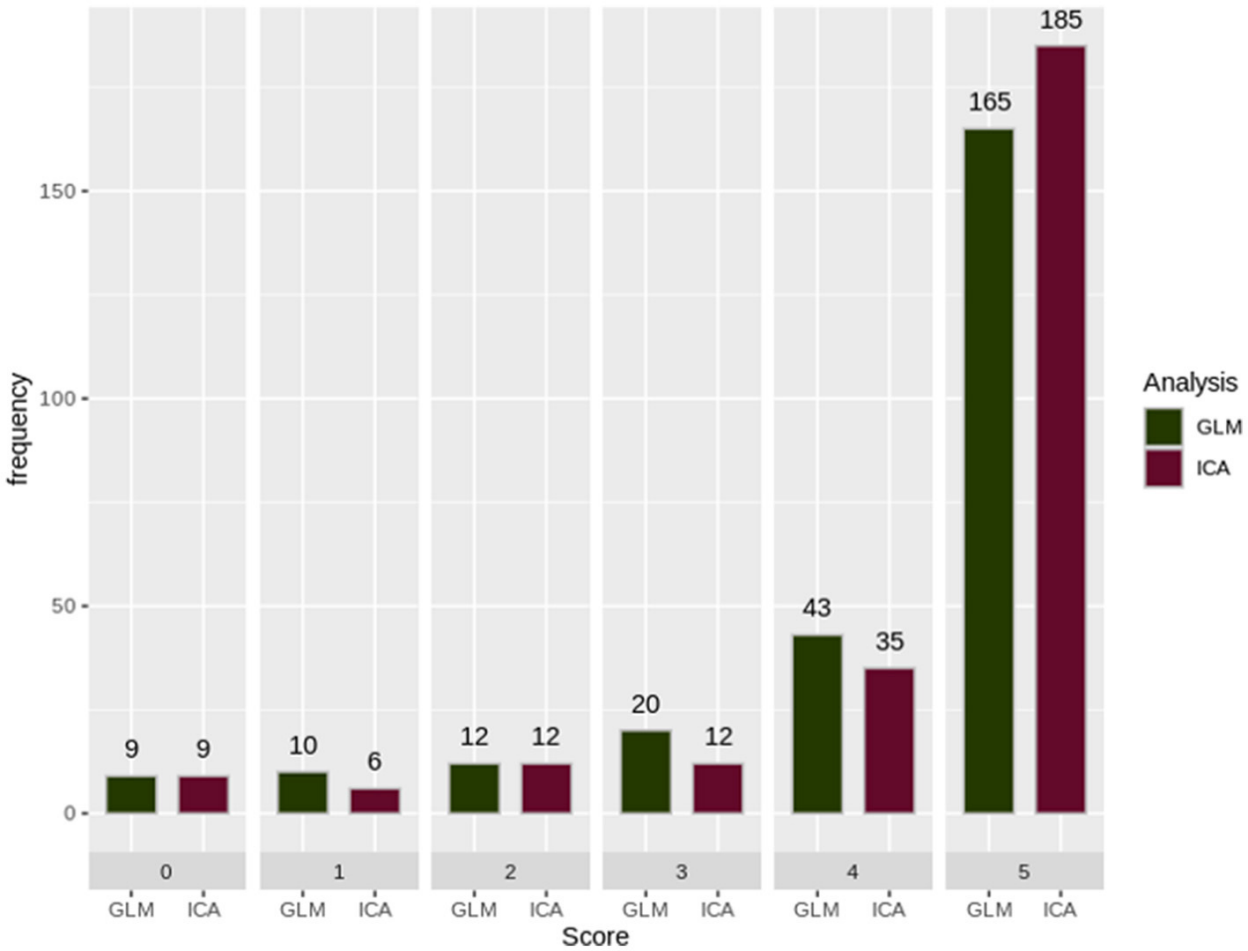
The sum of the scorings for each analysis technique. GLM, General Linear Model; ICA, Independent Component Analysis. As can be observed, GLM presented a motion-related artifact that interfered with the results, making them unreliable. ICA was able to differentiate and split these two signal sources into different components producing a reliable map. Images are in radiological orientation.

From a group of 60 patients and 20 controls, a total of 259 scans were collected and analyzed. For each scan, a GLM and an ICA map were chosen and were given a score. The total count of the scores is shown in Figure 3. As can be observed, both techniques performed fairly well. A paired *t*-test statistical analysis for the 60 scans of the control group showed no statistically significant different mean between ICA and GLM analysis techniques (*p* = 0.2425; mean of the differences = 0.1). The paired *t*-test of the 199 scans of the patients showed a statistically significant different mean (*p* = 0.002767; mean of the differences = 0.171), with ICA analysis techniques showing a greater mean value than the GLM technique. For Group 1, with 69 scans, ICA analysis scores showed a statistically significant greater mean than the GLM analysis scores (*p* = 0.0237; mean of the differences = 0.1594). For Group 2, with 130 scans, the ICA analysis scores displayed a statistically significant greater mean when compared to the GLM analysis scores (*p* = 0.01801; mean of differences = 0.1769).

**Figure 4:**
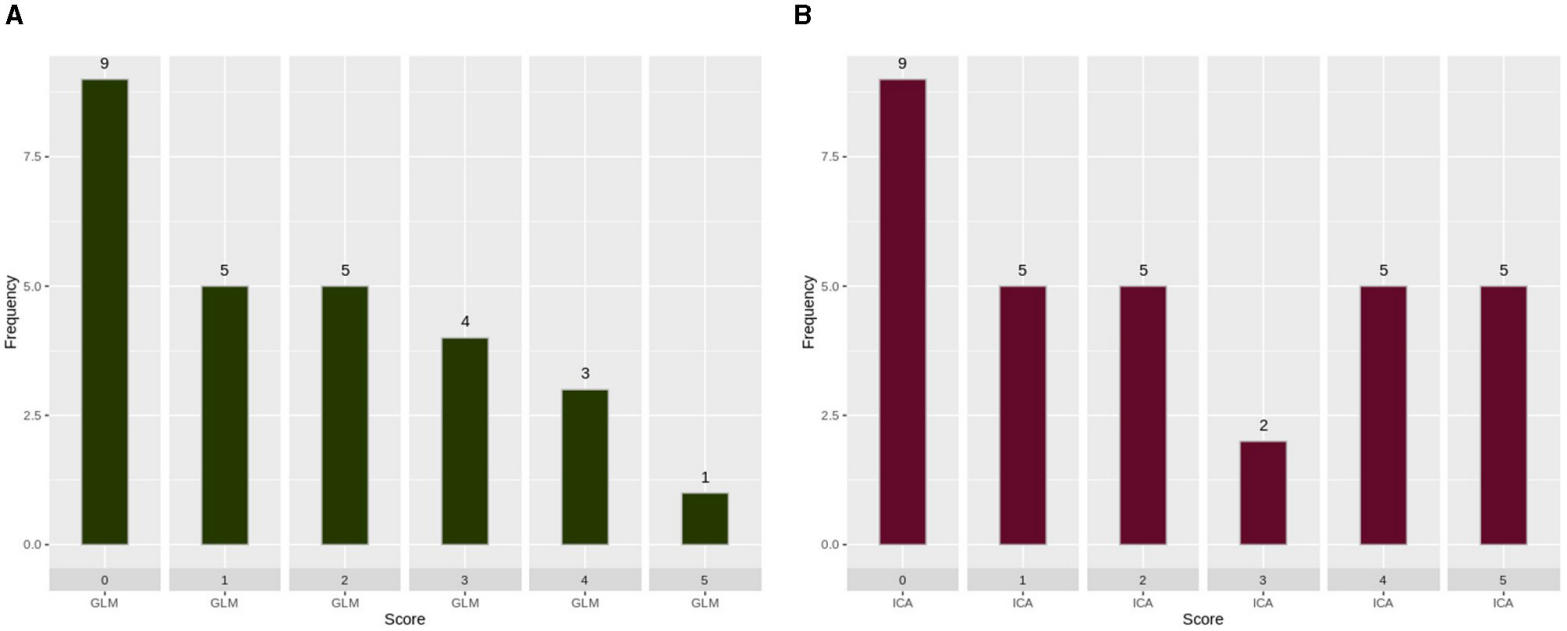
Scoring of each analysis technique when the other technique produced unreliable results. **(A)** GLM scoring when the score of ICA analysis was 0, 1, or 2. **(B)** ICA scoring when the score of GLM analysis was 0, 1, or 2. GLM, General Linear Model; ICA, Independent Component Analysis. As can be observed, ICA merged in a single component the activation with an artifact source resulting in an unreliable map. The ICA time series is far from the task time series. GLM produced a reliable map showing that the language areas were activated according to the task. Images are in radiological orientation.

A histogram was used to evaluate the differences and complementary nature of the two analysis techniques, as shown in Figure 4. In Figure 4A, we extracted scans (27 scans) in which the ICA technique scores were unreliable (scores of “0,” “1,” or “2”), and we presented the score of the GLM technique. Similarly, in Figure 4B, we extracted scans (31 scans) in which the GLM technique scores were unreliable (scores of “0,” “1,” or “2”), and presented the score of the ICA technique.

**Figure 5:**
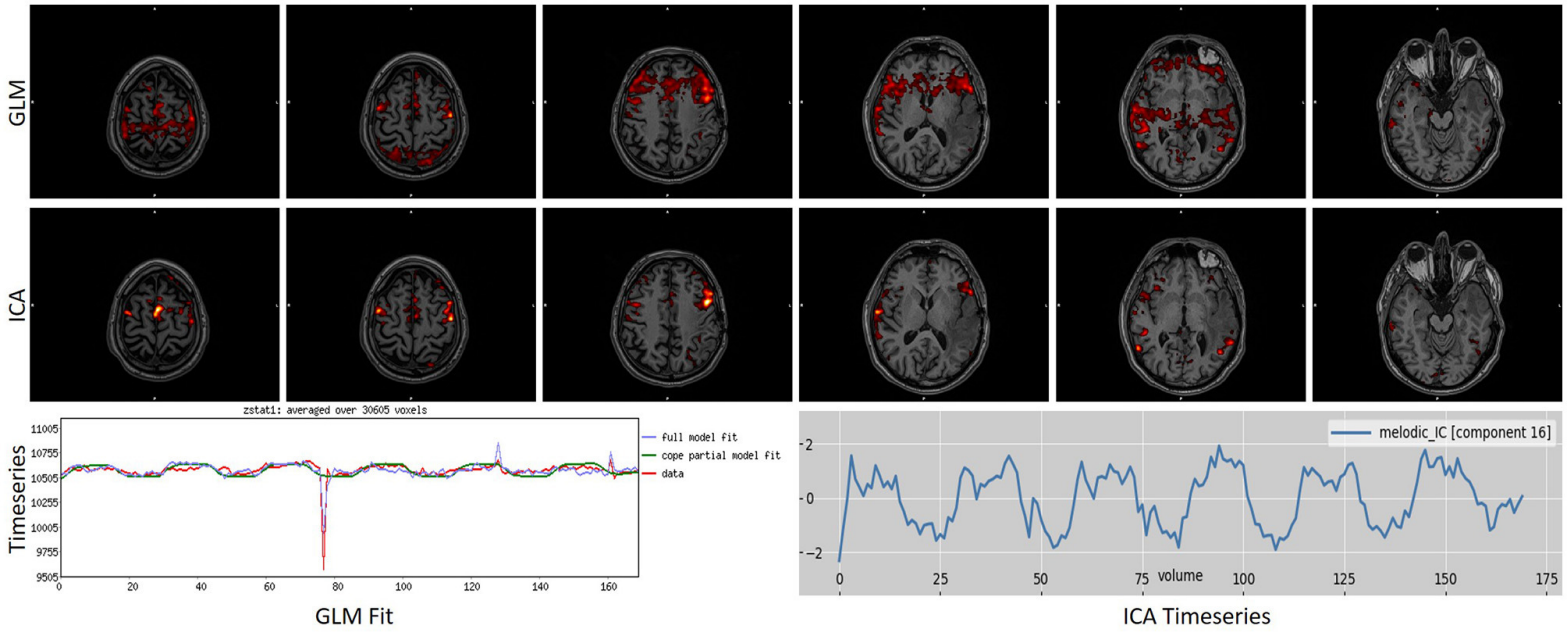
Example of a task that scored “1” in GLM analysis while scoring a “5” in ICA analysis. As can be observed, GLM presented a motion-related artifact that interfered with the results, making them unreliable. ICA was able to differentiate and split these two signal sources into different components, producing a reliable map. Images are in radiological orientation.

**Figure 6:**
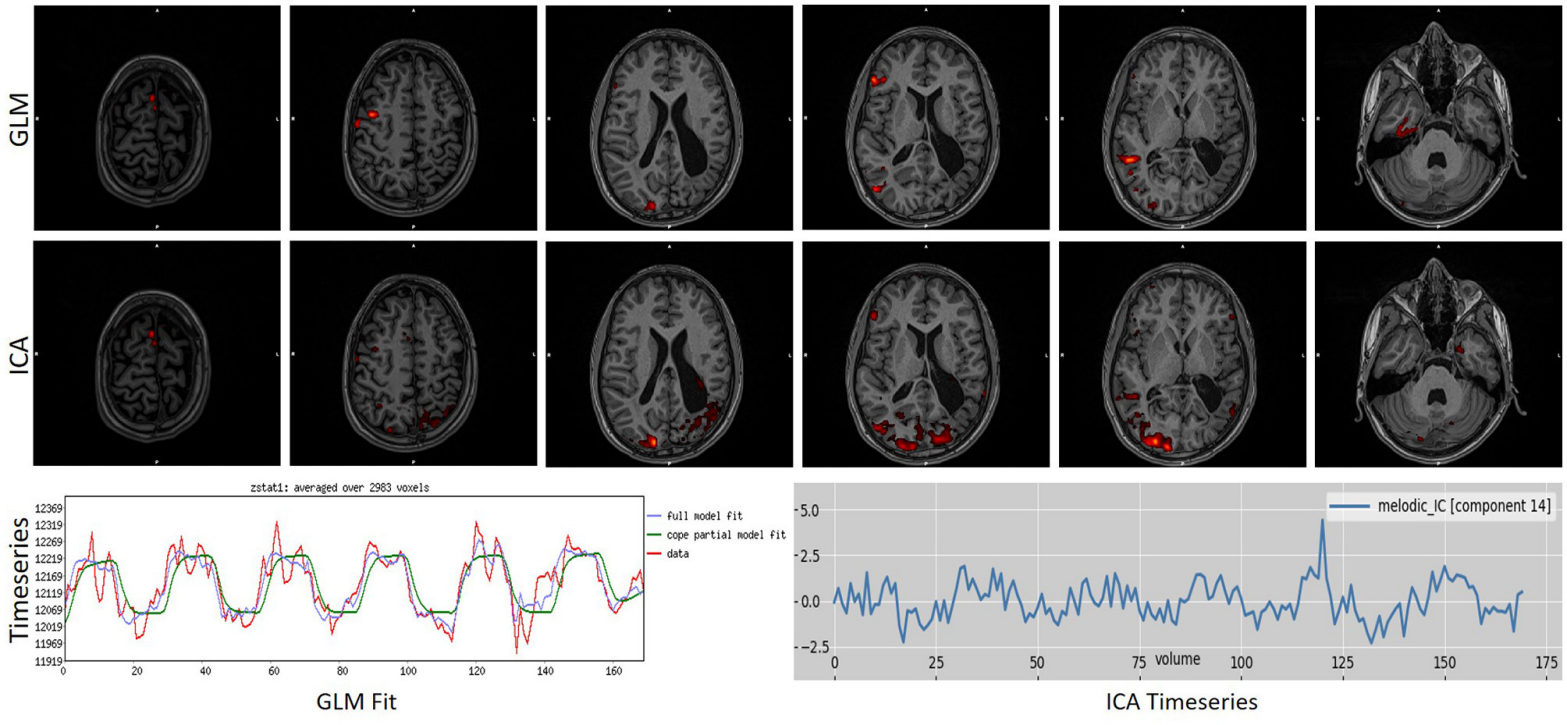
Example of a task that scored “1” in ICA analysis while scoring a “4” in GLM analysis. As can be observed, ICA merged in a single component the activation with an artifact source resulting in an unreliable map. The ICA time series is far from the task time series. GLM produced a reliable map showing that the language areas were activated according to the task. Images are in radiological orientation.

To further underline the different results between the two techniques, we created Figures 5, 6. In Figure 5, an example is presented where the GLM technique performed poorly, with a score of “1” while the ICA technique produced reliable results, with a score of “5.” Figure 6 presents the only scan where the ICA technique performed poorly, with a score of “2,” while the GLM technique performed excellently, with a score of “5.”

## 4. Discussion

In clinical practice, task-based fMRI has proven to be an essential examination prior to brain surgery. Nevertheless, there are many factors during the examination that can vary the results, frequently casting the scan session invalid. Such factors can be motion artifacts, notably when motion is correlated with the task, scanner artifacts, subjects' performance, and their neuropsychological state. In addition, physiological factors also play a key role in the results, such as the difference in the hemodynamic response of different brain regions, the effect of tumor or long-lasting epilepsy in the brain, and the effects of brain aging (15, 27).

Currently, the most widespread statistical analysis technique in use is the GLM. Although it is the only technique in medically approved software and it has been validated extensively, its intrinsic assumptions of linearity and the applied regressions do not allow for the abovementioned factors to be taken into account satisfactorily. Other shortcomings are that, even though a single hemodynamic response model was utilized for the whole brain, the motion parameters might not resolve the artifacts when motion is in line with the task, or the subject's performance and psychological state are not known to the model (14).

In this study, we sought an alternative to GLM that can be at least as reliable as GLM when conditions are ideal but can outperform it when not. ICA is a data-driven technique that can potentially overcome some of the abovementioned shortcomings. ICA splits the signal into spatial components that are statistically independent, which can later be identified as signal components/networks or noise components. The goal of the study was to identify if the activation of the task can be found among the signal components reliably and independently of other signal sources.

In fMRI data analysis, there are many considerations that can alter the statistical maps quantitatively and qualitatively. Pre-processing analysis techniques and parameters may significantly affect the results. As such, we utilized a common pipeline for all subjects, and both GLM and ICA techniques were fed with the same datasets. Since software packages may also influence the results, we employed the same software for both analysis techniques, namely FSL. Statistical thresholding of the maps is one of the immersive considerations in fMRI. To overcome this, we employed a methodology that included a variety of thresholds of the same statistical map and two fMRI expert clinicians who chose the final version of the map for each session blinded to what technique produced each map (15, 27, 28).

However, factors such as motion artifact and physiological noise that were not removed during pre-processing, as well as subjects' neuropsychological state and cooperation, were left to the GLM and ICA techniques to handle, because of which no subjects or sessions were excluded for any reason to assess the ability of each technique unbiased.

Following that, we included three groups of subjects. A control group of 20 subjects (60 scans) that, theoretically, should have performed well, and two patients groups: Group 1 with 38 patients with either congenital or early-life lesions (130 scans), although they usually cooperate well and their fMRI activations are often atypical, and Group 2 with 22 patients (69 scans) with high-grade pathologies that they, often, do not cooperate well due to their neuropsychological state [disease-specific and/or anxiety (27)]. Further, the acquisitions consisted of three fMRI language tasks for each subject that activate a spatially complicated statistical map with six language critical regions throughout the brain that includes frontal, temporal, parietal, occipital, median regions and, in some cases, deep brain structures (18). This allows us to evaluate the two techniques in maps with clusters in distant regions that may be affected or not by the brain disease and/or may differ in hemodynamic properties, as has been extensively suggested (29).

Statistical comparisons among the two techniques revealed: (a) no statistically significant difference between ICA and GLM in the control group, (b) a statistically significant greater mean value for ICA compared to GLM in the patient group when both Group 1 and Group 2 were included, (c) a statistically significant greater mean value for ICA compared to GLM in Group 1, and (d) a statistically significant greater mean value for ICA compared to GLM in Group 2. This further endorsed our hypothesis that the two techniques can perform similarly in the control group while ICA can outperform GLM in the patient group. It should be noted that both techniques performed satisfactorily well for clinical needs.

Even if not shown in the current study, ICA analysis revealed networks that were not linked to the language activation network either in their spatial map or in their time course. This further strengthens the assumption that many complex neuropsychological tests have suggested that brain dynamics are far more complex than simple on/off activations (15).

In Figure 4, the complementary nature of the two techniques can be observed. In this figure, the performance of each technique being assessed in cases where the other technique has failed is presented. Figure 4A shows the performance of GLM in the 27 scans in which ICA had poor performance. Similarly, Figure 4B shows the performance of ICA in the 31 scans in which GLM had poor performance. The nine scans that had scores of zero are the same scans for both techniques, so we believe that the subjects did not perform the task during those scans. It is evident that, by utilizing both techniques, the failed fMRI sessions can be minimized to 19 instead of 27 and 31 for each technique, respectively.

In most of the scans where GLM failed, ICA succeeded; the ICA component's time series was not as correlated with the task as in other scans. This can be explained by the inability of the subject to perform the task in a portion of the scan, which, in turn, can explain the reason GLM failed to produce reliable activation maps. In other scans, GLM failed due to motion parameters being correlated with the task. ICA succeeded in some of them, while in others, it could not differentiate, producing a map similar to GLM but an unreliable activation map. However, when ICA failed to produce a reliable activation map while GLM succeeded, we found that the maps had been either split into multiple components or had been merged with noise into a single component. This may change if we perform ICA analysis with different parameters for these scans, such as the number of components to be produced, but this was outside the scope of this study as we intended to assess the performance with the default parameters of each technique.

In literature, ICA has been established in fMRI studies as a reliable technique to extract network activations, although it has been mainly utilized in resting-state fMRI. Some studies in task-based fMRI have demonstrated that ICA is able to reveal networks and interactions beyond the GLM map activations (30–33). Although these studies are investigating group ICA analysis, networks of opposite activations and regions beyond the language networks were observed in our cohort as well. In another study, group ICA was able to differentiate activations even in multi-task fMRI designs (34). Ahmed et al. were able to differentiate the variability across trials, sessions, subjects, and brain areas using the ICA decomposition (35). In some recent studies by Boerwinkle et al. (36) ICA has been utilized successfully in single-subject resting-state fMRI analysis to reveal the epileptic network. They managed to validate the existence of the network in surgical cases as well (37, 38).

These studies demonstrate the advantages of group ICA to reveal more subtle activations and differences among populations over group GLM in complex neuropsychological fMRI experiments. In our study, ICA has been proven capable of extracting a complex brain network of language processes together with their time course at the single subject level. As such, we believe that ICA can offer a new perspective on brain dynamics and interactions in fMRI spatial maps, as well as their corresponding time courses. This can be applicable to group ICA and single-subject ICA, facilitating the integration of more complex neuropsychological tests into clinical translation.

To the best of our knowledge, the only study to perform single-subject ICA in task-based fMRI was by Tie et al. (39). Even if the focus of their study was group ICA analysis, they also reported single-subject ICA results from back-reconstructed maps of group ICA components. They found very high similarity scores of their ICA maps with the GLM results, similar to ours. In our study, we included a bigger cohort of controls (20 subjects), and we also included a group of patients (60 subjects; 38 Group 1 and 22 Group 2) for a total of 259 scans. However, more importantly, we chose to focus on methods that can be applied in single subjects separately without the need for a group analysis to prove the ability of ICA in clinical settings.

## 5. Limitations and future perspectives

There are some limitations in the current study that we would like to point out. Most important, the lack of neuropsychological assessments in our cohort limited the analysis we could perform in the study. Moreover, the number of subjects, although higher than any previous study and enough for statistical comparisons, is still not enough to drive ICA into clinical practice. More studies with similar results and larger and more diverse cohorts need to take place. From a methodological point of view, we believe that both GLM and ICA could perform better if parameters from each technique could be adjusted for each subject instead of taking the default values for all the scans. As such, another study could potentially compare these two techniques but with optimized parameters for each scan. Another methodological limitation of our study is the expert's evaluation of the language maps. This methodology implies objectivity, which may not always be present and, as such, may introduce some biases in the analysis. Though we chose this methodology over the alternative of a similarity score, as with the second, (a) we lost the ability to differentiate which technique performed better, and (b) we could potentially have high similarity scores even in unreliable language maps.

In our study, frequently, we found more ICA signal components that were not the activation of the task. A future study could potentially focus on these components to interpret their appearance and/or contribution to language processes.

To conclude, we need to emphasize the advancement in MRI technologies over the past few years that lead to acquiring high-quality data in time as well as in space. This allowed us to acquire fMRI data of 170 data points in a 5-min acquisition with an isotropic voxel size of 2 mm with full-head coverage. This is crucial for the successful implementation of the ICA methodology, as the fewer the data points, the faster the algorithms fall to overfitting. As such, a future study could focus on identifying the lower limit of fMRI data points that are needed during the acquisition for the successful ICA implementation in task-based fMRI.

## 6. Conclusion

In this retrospective study, we performed two analysis techniques—GLM and ICA—in language task-based fMRI data to assess the ICA technique's ability to perform well in clinical settings. For that reason, no exclusion criteria were set, and all 60 subjects that performed fMRI language were included in the study together with 20 healthy controls, resulting in 259 scans. ICA was able to perform as well as GLM in our control cohort while performing statistically better than GLM in patients independent of the underlying pathology. We demonstrated that ICA could extract the language map reliably in 232 out of 259 scans, while GLM extracted a reliable map in 228 scans. Notably, with the combination of both techniques, 240 scans could potentially produce reliable activations, improving the sensitivity of task-based fMRI in general. As such, we propose that, in practice, the implementation of both techniques in clinical settings significantly optimizes the sensitivity of task-based fMRI.

## Data availability statement

Anonymized data supporting the conclusions of this article can be made available if requested.

## Ethics statement

Ethical review and approval was not required for the study on human participants in accordance with the local legislation and institutional requirements. The patients/participants provided their written informed consent to participate in this study.

## Author contributions

KGk is the primary author being responsible of all the analysis and methods being described as well as the text writing of the manuscript. KGa provided the medical background necessary and together with NK evaluated the maps. IK provided the mathematical and theoretical background of the methodologies. AC and BZ as well as KGa involved in the data acquisition and provided the patient groups. All procedures, methodologies, results and, writing performed under the supervision and guidance of GM. All authors contributed to the article and approved the submitted version.
